# SMS text messaging to measure working time: the design of a time use study among general practitioners

**DOI:** 10.1186/s12913-018-2926-z

**Published:** 2018-02-20

**Authors:** Daniël van Hassel, Lud van der Velden, Dinny de Bakker, Ronald Batenburg

**Affiliations:** 1 NIVEL, Netherlands Institute for Health Services Research, P.O. Box 1568, 3500 BN Utrecht, The Netherlands; 2 CAOP, P.O. Box 556, 2501 CN The Hague, The Netherlands; 3 Tranzo, Scientific Centre for Transformation in Care and Welfare, Tilburg University, P.O. Box 90153, 5000 LE Tilburg, The Netherlands; 4 Department of Sociology, Radboud University Nijmegen, P.O. Box 9104, 6500 HE Nijmegen, The Netherlands

**Keywords:** Data collection, Text message (SMS), Workload data, Use of time, Working time, General practitioners, Health workforce planning

## Abstract

**Background:**

Measuring the working hours of general practitioners (GPs) is an important but complex task due to the effects of bias related to self-reporting, recall, and stress. In this paper we describe the deployment, feasibility, and implementation of an innovative method for measuring, in real time, GPs’ working time, plus the response to the study.

**Methods:**

A Short Message Service (SMS) application was developed which sent messages at random to GPs during their working week. Approximately nineteen GPs participated each week during a period of 57 weeks. The text messages asked if GPs were doing activities related to patients, directly, indirectly, or not at all, at the moment of sending. Participants were requested to reply by SMS.

**Results:**

Approximately 27,000 messages were sent to 1051 GPs over more than one year. The SMS system was functioning 99.9% of the time. GPs replied to 94% of all the messages sent. Only a few participants dropped out of the study. The data was available in real time enabling the researchers to monitor the response and overall quality of the data each day.

**Conclusions:**

The SMS method offers advantages over other instruments of measurement because it allows a better response, ease of use and avoids recall bias. This makes it a feasible method to collect valid data about GPs working time.

**Electronic supplementary material:**

The online version of this article (10.1186/s12913-018-2926-z) contains supplementary material, which is available to authorized users.

## Background

The use of one’s working time plays a pivotal role in health workforce planning aimed at maintaining, or restoring, a balance between the supply and demand for care provided by doctors. How many hours doctors actually work, related to their patients or not, is critical for assessing the supply that is available and needed in the future. This applies particularly to general practitioners (GPs) who function as gatekeepers in many health care systems and who play a central role in the access to care [1]. An important challenge, from a research perspective, is how to achieve a valid, reliable and accommodating method of measuring GPs’ working time. GP care is provided around the clock in most countries, so a measurement instrument to determine the working hours and use of time by GPs needs to be applied at any moment of a day or week. At the same time, many GPs complain about increasing administrative tasks, hence the possibility of participating in time consuming research is very limited, even though they realize how important this is.

There is currently an awareness of the availability, reliability and feasibility of methods to research the use of working time. But very little is known about methods or tools that can be applied to measure the working time among health care professionals such as GPs. A general rule is that these methods should match the setting it is applied to. This will depend on the target group and the type or level of activities being measured.

The dominant methods used to measure working time are observations, surveys and diaries. *Observational research* is considered to be a reliable research technique [2–4], but it is difficult and costly to apply on a large scale. This is particularly true when studying large numbers of work settings such as small general practices where observations of multiple respondents are only possible to a limited extent. The *survey method* is less costly but suffers from important disadvantages. Inaccuracy can occur due to the impact of recall bias and the socially desirable incentive for respondents to overestimate their working hours and specifically activities that are generally considered as a burden, such as administrative tasks. Clearly, these limitations also apply to GPs the subject of our study. Furthermore, it appears increasingly difficult to obtain sufficiently high response rates in survey research [5]. The *diary method* is considered as a good alternative to the observational method and is assessed as a reasonably reliable research technique [6]. However, the important limitations of keeping a conventional paper-based diary are the time and effort required from respondents and researchers [7]. There is, however, a growing number of communication and mobile devices that can reach respondents and send them simple signals or questions to measure their activities in real time. This type of research for sampling work activities has already been applied among nurses in 1995. These nurses carried a “bleep device” which provided a signal at random moments, after which they had to document their activity [6].

During the past few years, new communication techniques such as personal digital assistants (PDAs) [8–10], and smartphone apps [11] have been used increasingly as data collection tools. These tools yield more possibilities, but have their limitations as well. Devices such as PDAs require mobile internet availability and downloading of data afterwards which can result in possible data loss [8, 12]. The proper development, testing and design of smartphone apps yield relatively more expenses and are often dependent on the user having the latest version of the Android or iOS system [13]. However, Short Message Services (SMS) or text messaging lacks these compatibility or dependency problems as it is a relatively ‘old’ technology. It is a cheap and highly reliable type of mobile communication and is available on mobile devices of every kind. In almost all developed countries, nearly 100% of the population use a mobile phone and are able to send and receive SMS text messages. This makes SMS an attractive tool for measuring working time as it provides an easier and cheaper way to collect real time data from large populations. There are, however, few studies in which SMS have been used to measure the working time of certain populations. One of the few exceptions is a validity study of Brenner and colleagues [13] who applied SMS text messages to measure the use of time among university students. To our knowledge, there are no studies about the use of time in which SMS have been applied among health care professionals such as GPs.

We piloted an SMS-based measurement among 14 GPs to test if an SMS tool would be a proper instrument to measure their working hours in real time [14]. This achieved positive results. It was experienced as feasible by the respondents and delivered valid measurements. For example, a response was received on 96% of the activity messages sent and 66% of the messages sent was replied within 10 min. Furthermore, the respondents confirmed their answers on the activity messages after the week of SMS messages. Subsequently, we applied the design and SMS instrument on a larger scale in a project including more than 1000 Dutch GPs over a period of 14 months. In this paper, we describe the development and application of this real time measurement tool for conducting large-scale research into the use of working time. The research question that will be addressed is: “How could a real time measurement tool be developed; and what can we say about its feasibility and ease of implementation?”

## Methods

The target group of the SMS-based measurement of the use of working time were all active and employed GPs in the Netherlands. In 2013 the population concerned 11,075 GPs [15]. Two types of samples were designed:

1. A stratified sample of six types of GPs (male and female self-employed, salaried and locum GPs).
2. A sample of time points for sending these GPs a limited number of SMS messages on a daily basis during one week.

Considering this two-staged sampling approach, we aimed to include at least 1000 GPs in our study.

### Recruitment and planning

The data was collected from December 2012 to January 2014 in order to account for seasonal variability in GPs working time. This period was divided into seven consecutive sub-periods of two months for which batches of participants were recruited to participate in one of the weeks (Table 1). For every batch, a letter of invitation was sent to a stratified sample of approximately 500 to more than 800 GPs registered in the career database of the Netherlands institute for health services research (NIVEL). This database covers nearly all active GPs in the Netherlands and has been managed by NIVEL since 1974 [15]. The invitation letter contained a personal weblink to register for the time use study. Non-respondents received two reminders. The sampling and invitation mailing were conducted mostly two months prior to the start of every sub-period. The recruitment for the first batch started in October 2012 and for the final batch in September 2013. GPs could be included from the moment they received the invitation letter until the first month of a particular batch. In addition to the invitation letters, announcements were made in several GP media channels such as professional newsletters and websites. Our study was supported by several organizations from the field, such as Dutch GP associations and the Association for GP trainees and trainers.

**Table 1:** The seven batches for which GPs were invited in 14 months

	Recruitment participants in period	Start SMS weeks in period	Weeks of the year/SMS weeks	Number of invitations
Batch 1	October – December ‘12	December ‘12 - January ‘13	49 to 52 and 01 to 04	555
Batch 2	November ‘12-January ‘13	January–February ‘13	01 to 09	537
Batch 3	January – March ‘13	March–April ‘13	10 to 18	781
Batch 4	March – May ‘13	May–June ‘13	19 to 26	795
Batch 5	May – July ‘13	July–August ‘13	27 to 35	794
Batch 6	July – September ‘13	September–October ‘13	36 to 44	728
Batch 7	September – December ‘13	November ‘13 - January ‘14	45 to 01	838

### Subscribing for the research by completing surveys

Two surveys were hosted on a dedicated (NIVEL.nl) website that was launched for the time use study (Additional file 1). The content of the questionnaires can be found on pages 115–119 in our final report of the study which was published earlier [16]. The first survey was for GPs to log on prior to their allocated SMS week. They used the username and password received with the invitation letter or by a separate email for respondents who followed the open weblink in the announcements published in the media. Batches of GPs, approached during a specific sub-period, were asked to participate in a week, proposed at random, during the two-month period. When GPs were not available in the proposed week, a maximum of four other weeks were offered. In case participants were not available in these weeks either, they received a new username and password to log on to be allocated to another week in the time use study during the next sub-period (batch). Questions were asked in the pre-survey in order to ascertain the background of the respondents, for example their socio-demographic characteristics, employment position, or type of practice.

After GPs completed the week in which their use of time was measured, they were asked to complete a ‘post survey’ on the NIVEL time use research website. One of the questions in this survey was aimed at obtaining the experiences of GPs regarding the feasibility of participating in an SMS week. Participants were reminded to complete the post survey by SMS at the end of their SMS week and also by sending them a separate email. If they completed the post survey, they received a €10 gift voucher to compensate for their SMS costs and for replying to the SMS questions.

### The SMS application and the privacy of the respondents

A professional SMS service provider was sub-contracted in order to deploy, technically, the SMS measurement application. This company programmed the customised SMS application and was responsible for the daily sending and receiving of all SMS messages to and from the participating GPs during the period studied.

To ensure the privacy of the respondents, the researchers anonymised and coded all participants by a response number. Prior to each week in which their use of time was measured, the response numbers and phone numbers of the participants were sent in a protected and secured data file to the SMS service provider. The phone numbers were provided by the GPs to the research project as part of their consent - that is through the pre-survey with which they registered for the research. The SMS service provider sent a confirmation email to the researchers once they had received the data file. During the study the data received on the use of time was available for the researchers in real time on a website of the service provider. This website was secured and only accessible on the computers of the researchers by means of a username and password.

### Recording the activities of GPs by SMS: Activity messages

**Fig. 1:**
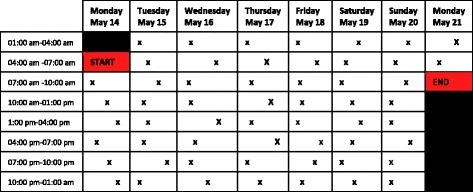
Time sampling frame with three hours’ time slots in which the activity messages were randomly scheduled

An important step was to design the messages needed to measure the working time of GPs. As described before, during one full week, all participating GPs were queried about their activities by sending SMS texts on a daily basis. The main type of SMS texts were designed as so-called *activity questions* sent at random moments within three hours’ time slots (Fig. 1, Additional file 2).

**Fig. 2:**
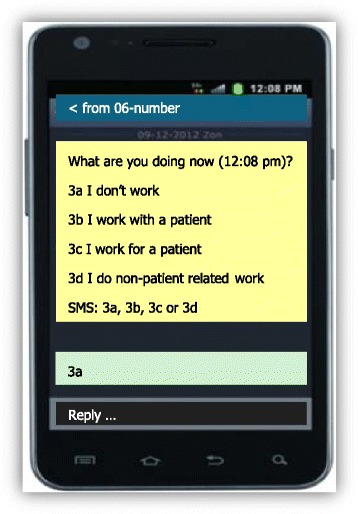
Screen shot of an activity message that was sent at 12:08 pm

The content of the activity messages was designed to be as clear and concise as possible in order to ensure they were as practical as possible, such that the respondent was able to reply without thinking too much. It was decided that the activity messages should include only one basic question: “What are you doing at [time]?” The display “[time]” contained the exact moment at which the SMS was scheduled and sent (Fig. 2). The response categories, presented below the question, could not contain too much detail, as it had to fit within the maximum size of a single SMS message. Hence only four concise response categories were presented for the question relating to “at this moment”. They were: (1) I’m not working; (2) I do direct patient-related work; (3) I do indirect patient-related work and; (4) I do work not related to patients. These four answer categories are comparable or can be compared by aggregation with other time use studies among health care professionals [17, 18].

To ensure the four categories were clearly understood and chosen properly by the participants, definitions were provided in a specific instruction that was made available for the respondents (see also Additional file 1).

The calculation of the average working hours of GPs based on the activity messages send is explained in Additional file 3.

### Additional types of messages to enhance their feasibility and the quality of data

Five additional types of messages were sent to improve the feasibility of the measurements of time use and to support the overall quality of the data.

#### Messages announcing the SMS week

Respondents were mostly allocated to their survey week over one month in advance. Therefore, for every GP their SMS week was announced by a separate message that was sent on Sunday at 4:00 pm, the day before the SMS week started. Half an hour following this message an additional SMS provided information about where to find instructions for the SMS week (Additional file 1). Furthermore, this message alerted the participants to the planning messages that would be sent out early in the morning every day.

#### Planning messages

**Fig. 3:**
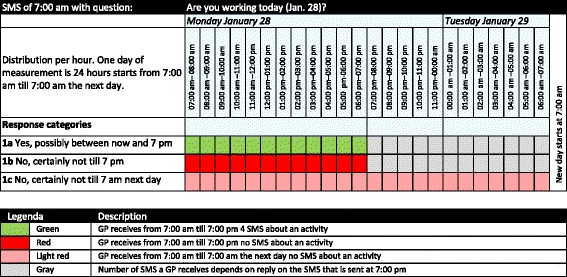
Day planning question and the consequence of the response categories for the sending of activity questions in the subsequent period

The messages that measured the actual use of time (‘activity messages’) were scheduled during a whole week, including all evenings, nights and weekend days. This ensured that many GPs would receive messages during significant parts of a week in which they were probably not working. At 7:00 am and 7:00 pm each day, GPs received a planning message asking if they were planning to work, or not to work, during the upcoming part of the day. If GPs indicated that they would not work, such as because they were off duty or on leave, then the system was designed not to send ‘activity messages’ in every three hours’ time slot during these periods of time. Figure 3 demonstrates the pattern of activity questions that were sent as a result of the response to the questions on the planning of the GP’s day. A confirmation SMS was returned after a participant responded to the planning questions.

#### Reminders

A time limit of 24 h was programmed to process and store a participants’ reply while at the same time, all respondents were instructed to reply to the activity and planning messages as soon as possible. A reminder was sent half an hour after the activity message to avoid recall bias as much as possible and support a quick response. This reminder SMS message contained a short text followed by the same question - what the participants were doing at the moment they received the reminded activity message.

#### Correcting messages

Invalid answers can be a major concern as they can bias the results [19]. Also, by using SMS, invalid answers can easily be entered by users, especially during a busy day. To try to prevent this, the system sent a correction message to the respondent in cases an invalid answer was replied on an activity message or planning message. For every time a participant replied with an invalid answer, the application sent an SMS to request a correct answer. Furthermore, if an unintended reply was entered, participants were able to correct their answers within 24 h, simply by sending a new SMS with the corrected answer.

#### Messages announcing the end of the SMS week

All the GPs participating received a ‘thank you and end of measurement’ message on Monday afternoon at the end of the week in which their working time was measured. Furthermore, this message was used to remind them of completing the survey following participation.

### Replying SMS messages using numbers

Participants had to reply to every pre-scheduled message with a response character and the number of the message. The number of the message concerned the number in a series of scheduled messages on a specific day (Table 2). The numbering started every day at 7:00 am. Numbering the SMS messages was required in order to identify the messages received and to attach them to the correct message that was sent. Only one answer was accepted by the application.

**Table 2:** Sending of different types of SMS messages for one day, including message number of a day

Message-number	Time (slot) scheduled	Type of message	Comments concerning sending of messages
1	07:00 am	Planning message: day	Always sent
2	07:00 am-10:00 am	Activity message; 1st time slot	Depends on day planning
	*07:30* am *−10:30* am	*Reminder; 1st* time slot	*If no reply within half an hour*
3	10:00 am −01:00 pm	Activity message; 2nd time slot	Depends on day planning
	*10:30* am *−01:30 pm*	*Reminder;* 2nd *time slot*	*If no reply within half an hour*
4	01:00 pm −04:00 pm	Activity message; 3th time slot	Depends on day planning
	*01:30* pm *−04:30 pm*	*Reminder; 3th* time slot	*If no reply within half an hour*
5	04:00 pm − 07:00 pm	Activity message; 4th time slot	Depends on day planning
	*04:30* pm *− 07:30 pm*	*Reminder; 4th* time slot	*If no reply within half an hour*
6	07:00 pm	Planning message: evening/night	Depends on day planning
7	07:00 pm-10:00 pm	Activity message; 5th time slot	Depends on day- or evening/night planning
	*07:30* pm *−10:30 pm*	*Reminder; 5th* time slot	*If no reply within half an hour*
8	10:00 pm −01:00 am	Activity message; 6th time slot	Depends on day- or evening/night planning
	*10:30* pm *−01:30* am	*Reminder; 6th* time slot	*If no reply within half an hour*
9	01:00 am −04:00 am	Activity message; 7th time slot	Depends on day- or evening/night planning
	*01:30* am *−04:30* am	*Reminder; 7th* time slot	*If no reply within half an hour*
10	04:00 am −07:00 am	Activity message; 8th time slot	Depends on day- or evening/night planning
	*04:30* am *−07:30* am	*Reminder; 8th* time slot	*If no reply within half an hour*

## Results

### The implementation and governance of the time use survey instrument based on SMS

Our SMS-application sent and received thousands of messages over the course of a year. The uptime of the system was nearly 100%. There was only one day in which no messages could be sent because of a breakdown in telecommunications which affected other services in the region as well. The SMS messages for that specific day were recalled manually and inserted into the database. During the whole period of fieldwork, the SMS data was processed and stored directly in one central database hosted by the service provider. Researchers were able to log on a secured website in order to download this data in real time in an Excel-file. If no reply from a participant was received or processed during 24 h then the application sent an SMS and email to the researchers. This notification, accompanied with the real time availability of the data, made it possible for the researchers to continuously monitor, all responses to the messages, and the overall quality of data. This meant that problems regarding the sending and receiving of the messages were noticed on time and solved by the researchers by contacting the respondents or the SMS provider, if necessary. Forty-two respondents stopped participating either during the survey week, or a substantial part of it, mostly because of personal circumstances. In these cases messages could be stopped by the researchers through the secured website of the SMS service provider.

At the start of the fieldwork some problems appeared regarding the unique short code ‘6565’ that was initially used by the application to send the messages. The service provider of some respondents blocked messages sent by short codes because of security or because the users had requested that pay-based messages be blocked. To avoid participants being charged additional costs, the short code was changed into a ‘regular’ Dutch mobile 06-number.

### The response of GPs to the invitation letters and media announcements

In total 5028 letters of invitation were sent to 4486 individual GPs, some receiving a letter more than once. Of these 782 (=response rate 17%) participated in at least one full SMS week. In addition to the letters of invitation, GPs were recruited through announcements made in several media. In total, 1051 GPs participated in one of the survey weeks, 44 of them participated twice. The dataset provided sufficient observations and power to calculate reliable average numbers of working hours for GPs, and, specifically for six subgroups of GPs (male and female self-employed, salaried and locum GPs). The composition of the participant group corresponded reasonably well with the stratified sample drawn from the NIVEL registration of GPs (Table 3). There were slightly more self-employed GPs and fewer GP locums included in the participant group compared to the GP sample. On average, 19 GPs participated in each week during the fieldwork period.

**Table 3:** Numbers and distribution of the participating’s (participants) and the invitations (invited GPs) by different background characteristics^a^

	Participating’s (participants)^b^				Invitations (participants invited)^c^			
	N		%		N		%	
Employment position								
Self-employed	642	(618)	58.6	(58.8)	2300	(2224)	45.7	(49.6)
Salaried	214	(203)	19.5	(19.3)	1027	(894)	20.4	(19.9)
Locum	239	(230)	21.8	(21.9)	1701	(1368)	33.8	(30.5)
Total	1095	(1051)	100.0	(100.0)	5028	(4486)	100.0	(100.0)
Gender								
Male	461	(438)	42.1	(41.7)	2380	(1877)	47.3	(41.8)
Female	634	(613)	57.9	(58.3)	2648	(2609)	52.7	(58.2)
Total	1095	(1051)	100.0	(100.0)	5028	(4486)	100.0	(100.0)
Age in years								
< 40	496	(478)	45.3	(45.5)	2266	(1930)	45.1	(43.0)
40 to 49	283	(265)	25.8	(25.2)	1340	(1229)	26.7	(27.4)
50 to 59	268	(260)	24.5	(24.7)	1129	(1046)	22.5	(23.3)
≥ 60	48	(48)	4.4	(4.6)	293	(281)	5.8	(6.3)
Total	1095	(1051)	100.0	(100.0)	5028	(4486)	100.0	(100.0)

^a^The sum of the percentages could deviate of 100% as a result of rounding

^b^This concerns the GPs who responded to the invitation letters and media announcements. There are 1095 participating’s as 44 GPs participated twice

^c^We took a stratified sample of the GP population with a different chance by employment position and gender. The background characteristics are based on the NIVEL registration of GPs and the pre-survey. There are 5028 invitations as some GPs were invited more than once

### SMS messages: Numbers and response

During the research period, the SMS service provider pre-programmed 61,320 activity messages for the 1051 individual GPs who had agreed to participate during a specific week. In total 26,675 (44%) activity messages were sent after the planning messages were replied to by the participants. As can be expected, activity messages were mostly *not* sent, upon the request of the participants, during evenings, nights and weekends.

The survey following participation was completed 972 times. In this survey GPs indicated they had some difficulties with the planning messages that were sent at 7 am in the morning including the weekend days:

*“Interesting study, it was nice to participate. But the SMS text messages I received at 7 am in the morning on my day off were not so pleasant…”*

*“An SMS at 7 am on a weekend day is too early for me.”*

However, in general they experienced a low burden and felt positive with regard to the type of survey and the SMS-based instrument in particular. They reported the messages did not disrupt their work too much, and they had no difficulties replying on the messages:

*“It was not difficult to participate. The SMS text messages did not disrupted me during my work.”*

*“Replying on the SMS text messages was very practical. It did not take too much time.”*

These experiences are also reflected in the response rate (94%) to the activity messages sent. Of all the activity messages, 51% was replied to within ten minutes and 80% within one hour. The response rate and response time was higher during regular weekdays compared to the response on weekend days and holidays. For example on weekdays the response rate was approximately 96% and more than 50% of the activity messages was replied within 10 min. On weekend days and holidays this was approximately 90% and 40%.

GPs received an overview of their personal SMS response pattern after the survey week. This resulted in ten to 20 emails from participants that suggested some corrections of their answers.

## Discussion

Our study sought to describe the deployment, feasibility and ease of implementation of an SMS-based time use method applied among Dutch GPs. The research was conducted over a period of 57 consecutive weeks in which 1051 individual GPs participated, an average of almost 20 participants per week. During the survey week, the participants received multiple messages on a daily basis which questioned them about their activities at random selected points in time (in blocks of three hours).

The study showed that by applying the SMS instrument of time use it is possible to collect valid and rich data in a user friendly manner among a large group of respondents – in this case Dutch GPs. The measurement tool was shown, in particular, to be a feasible instrument for collecting data about the use of working time among different groups of GPs and for a limited number of activities. The high response rate to all the SMS messages sent, and the positive reactions of GPs after participation, are good indicators of its success.

There are several factors which contributed to the success of this study. Firstly, a lot of effort was invested in the recruitment of the participants. More than 5000 invitation letters were sent with support from professional organisations such as the Dutch GP association. In addition, a significant number of GPs subscribed to the research not directly invited. Secondly, although only a relatively small allowance for GPs was offered to compensate for their SMS costs - a gift voucher of €10 - this was sufficient for their recruitment. Thirdly, the design of the SMS instrument was driven by the aim to reduce the burden on the participants as much as possible. GPs could easily reply on the SMS messages by simply entering a number and one multi choice letter. Additionally, participants could indicate when they were not working during a particular part of the day, avoiding their being interrupted unnecessarily with so-called activity messages. Finally, the SMS service provider developed an SMS application which ideally suited the aim of the research which was to measure the working time of GPs in a valid and user-friendly manner. This system managed to measure the working time of different GPs at all times during one year with a 99.9% uptime.

The use of the SMS service provider, who was responsible for sending and receiving the SMS messages and for processing the data, did involve considerable costs. In particular there were relatively high costs for the development of an SMS-application which suited the aim of our study. These start-up costs would be considerably lower, however, were this research to be repeated in collaboration with the same SMS service provider. Additionally, limited material costs were involved as almost everybody has a mobile phone at their disposal to send SMS messages [20] and the sending of SMS is becoming cheaper. A paper-based diary method would have involved considerably higher material costs for paper and postage. It is also very likely that the costs of the SMS instrument are lower compared to observations on large scale as these would have resulted in the higher costs and greater effort required for observing the GPs [3].

The design of the method was aimed at GPs in the Netherlands. Nonetheless, this instrument could be applied in other countries as well, because of the broad availability of mobile phones even in low- and middle income countries [8]. The method could, with some adaptations, also be applied to other target groups. For instance, just as with GPs, it is important to gain insight into activities, related to patients or not, of other health care professions which can support manpower planning [21]. Even professional groups not involved in health care such as bus drivers or construction workers are a possible target group, although here the response categories would be defined differently. It would be necessary to adapt other parts of the design, depending on the working time of which target group will be measured. For example, it may not be necessary to measure the use of time 24 h a day, seven days a week, when out of office hours are not relevant. Yet, at the same time, it would be possible to perform more measurements per respondent during a day and include a lower number of participants.

The design of the SMS-based study described is adequate to obtain data from some general activities. A single SMS provides enough memory for a simple question and a limited number of response categories, but the obtained information is more limited compared to many other methods. For example Sinsky et al. developed a time and motion study in which the allocation of time on 12 activities of physicians was recorded by observers. The observers coded among others what the physicians were doing, where they were doing it and with whom they were engaged [22]. Yet, with some adaptations to the design the SMS tool can also provide more detailed information about certain activities, for example when it concerns the time spent on specific indirect patient-related activities such as travelling to a patient or the registration of patient data. An option is to send two or more types of questions with a smart allocation of questions for different respondents. One could also think of two or more consecutive questions based on the response to the first question.

### Limitations

There are some limitations to this study. Firstly, the 56 SMS measurements per week for a GP provide only a broad estimation of the time use for one GP. The method is only appropriate when more participants are included, because this yields an increasing number of measurements for a target group. An accurate calculation of the average working hours can then be made.

Secondly, in our study design we chose to measure the working hours of more than 1000 GPs over a year. We chose this time period and included this number of respondents to account for seasonal variation and to enable a proper measurement of working time for different types of GPs. This shows that it takes time to collect data and recruit participants. Future studies have to provide more insight into the change of confidence intervals of the mean working hours if data is collected over a shorter period among fewer participants and if the frequency of measurements per participant is adapted.

Thirdly, the SMS application could have imposed a burden upon GPs by interrupting their daily use of their phone. We had no direct indications this actually happened. However, some participants indicated that the planning questions which were sent every morning at 7:00 am were disruptive, particularly during weekends. This was necessary for our target group as they could also work during these irregular moments. We did account for this by informing the GPs about these messages at the start of the SMS week and by recommending that they turn off their cell phones and respond later if they did not want to be disturbed. These experiences had limited implications for the results, however, because responses during weekends and holidays were lower, but still acceptable.

Finally, we applied SMS as a real time data collection tool as it provides benefits such as the broad use of mobile phones and the availability of SMS services. Yet modern research tools are becoming available more often, yielding new possibilities for gaining insight into highly detailed activities. For example, one might think of an app in which an activity can be selected via different menus and where additional questions are asked by a pop-up window [11]. Even so, we suggest that the study design described in this paper could also be used even when these kinds of data collection tools are preferred.

## Conclusion

The SMS method appears to be a feasible method to collect real time data about the working hours of large populations and different subgroups, during one year. The method has potential for broader application in other countries and among other target groups, possibly with some small adaptations. Further research has to provide more insight into the question of to what extent the real time measurement tool could be applied reliably among a smaller number of respondents during a shorter period of time.

## Additional files

Additional file 1: Website with surveys and instructions for the SMS week. (DOCX 16 kb)

Additional file 2: Time sampling frame applied for sending activity messages to GPs, during one week. (DOCX 16 kb)

Additional file 3: Calculating the working hours based on SMS. (DOCX 16 kb)

## Abbreviations

GP: General practitioner
NIVEL: Netherlands institute for health services research
PDA: Personal digital assistant
SMS: Short message service
