# Supplementary material for: SMS text messaging to measure working time: the design of a time use study among general practitioners
Source: BMC Health Serv Res. 2018 Feb 20;18:131. doi: 10.1186/s12913-018-2926-z (PMC5819672; doi:10.1186/s12913-018-2926-z)
Supplement: Supplementary file 1 — Website with surveys and instructions for the SMS week. (DOCX 16 kb) [file 12913_2018_2926_MOESM1_ESM.docx]

At the start of our research, we launched a website on which the respondents could log on the surveys to subscribe and unsubscribe for the research. Furthermore, the website provided respondents with background information about our research and an instruction relating to the SMS week. This instruction contained information about the messages and how they had to reply. A shorter version of this instruction was also available and included with the invitation letters which we sent to the participants. GPs could easily use this instruction during the SMS week in order to consult the definitions of the response categories.
