# Supplementary material for: SMS text messaging to measure working time: the design of a time use study among general practitioners
Source: BMC Health Serv Res. 2018 Feb 20;18:131. doi: 10.1186/s12913-018-2926-z (PMC5819672; doi:10.1186/s12913-018-2926-z)
Supplement: Supplementary file 2 — Time sampling frame applied for sending activity messages to GPs, during one week. (DOCX 16 kb) [file 12913_2018_2926_MOESM2_ESM.docx]

The goal was to design an SMS survey of time use that balanced accuracy and feasibility to enable us to estimate the working hours of GPs on an aggregated level. An important design decision concerned the frequency and pattern of the messages for every GP. More messages for every participant would increase the accuracy of the measurements, but, probably, also the number of drop outs. Furthermore, it would demotivate participants if the messages came up too soon. Keeping these considerations in mind, we decided to measure the working time of every GP during one week and to divide this week into three hours’ time slots (figure 1). An SMS message was scheduled at a unique random moment for every GP within each of these time slots. A minimal interval of five minutes was set between two measurements in two consecutive time slots. Every GP received a maximum of (24 hours/three hours=) eight messages a day measuring their activity. Every GP was scheduled to receive 56 messages per week (eight messages a day*seven days). We assessed this prior to the study as an acceptable number of messages for the participants, during this period of time.
