# Supplementary material for: SMS text messaging to measure working time: the design of a time use study among general practitioners
Source: BMC Health Serv Res. 2018 Feb 20;18:131. doi: 10.1186/s12913-018-2926-z (PMC5819672; doi:10.1186/s12913-018-2926-z)
Supplement: Supplementary file 3 — Calculating the working hours based on SMS. (DOCX 16 kb) [file 12913_2018_2926_MOESM3_ESM.docx]

Working hours were calculated by multiplying the replies to the questions about activities by three as these were the time slots in which the messages were sent during the week. For example a GP who replied 13 times one of the answers, b, c or d (“At this moment I am working; (b) directly; (c) indirectly, or; (d) not directly or indirectly with patients”) would work 13 x 3=39 hours. This provides a broad estimate of every GP’s working week. However, the method is appropriate when more participants are included, because this results in an increasing number of measurements for a target group as a whole. An accurate calculation of the average working hours can then be made.
